# OCT-Angiography reveals reduced vessel density in the deep retinal plexus of CADASIL patients

**DOI:** 10.1038/s41598-018-26475-5

**Published:** 2018-05-25

**Authors:** Pieter Nelis, Ilka Kleffner, Matthias C. Burg, Christoph R. Clemens, Maged Alnawaiseh, Jeremias Motte, Martin Marziniak, Nicole Eter, Florian Alten

**Affiliations:** 1 Department of Ophthalmology, University of Muenster Medical Center, Muenster, Germany; 2 Department of Neurology, University of Muenster Medical Center, Muenster, Germany; 3 Department of Clinical Radiology, University of Muenster Medical Center, Muenster, Germany; 4 Department of Neurology, Ruhr University Bochum, Bochum, Germany; 5 Department of Neurology, kbo-Isar-Amper-Klinikum München-Ost, Muenchen, Germany

## Abstract

Optical coherence tomography angiography (OCT-A) represents the most recent tool in ophthalmic imaging. It allows for a non-invasive, depth-selective and quantitative visualization of blood flow in central retinal vessels and it has an enormous diagnostic potential not only in ophthalmology but also with regards to neurologic and systemic diseases. Cerebral autosomal dominant arteriopathy with subcortical infarcts and leukoencephalopathy (CADASIL) is a hereditary vascular small-vessel disease caused by Notch3 mutations and represents the most common form of hereditary stroke disorder. In this study, CADASIL patients prospectively underwent OCT-A imaging to evaluate retinal and choriocapillaris blood flow as well as blood flow at the optic nerve head. The vessel density of the macular region and the size of the foveal avascular zone in the superficial and deep retinal plexus were determined as well as the vessel density at the optic nerve head and in the choriocapillaris. Additionally, cerebral magnetic resonance images were evaluated. The main finding was that vessel density of the deep retinal plexus was significantly decreased in CADASIL patients compared to healthy controls which may reflect pericyte dysfunction in retinal capillaries.

## Introduction

Cerebral autosomal dominant arteriopathy with subcortical infarcts and leukoencephalopathy (CADASIL) is a hereditary vascular small-vessel disease caused by Notch3 mutations^1^. It is distinguished from other vascular disorders by the characteristic accumulation of granular osmiophilic material in brain vasculature^2^. Ischemic strokes are the most frequent manifestations in CADASIL, occurring in up to 85% of patients at a mean age of 49 years mostly in the absence of common vascular risk factors^3^.

Besides other neurological conditions like migraine, apathy, cognitive impairment and mood disturbances, CADASIL is clinically also characterized by numerous ophthalmologic findings^4–6^. In 2014, our group used high-resolution retinal imaging including spectral-domain optical coherence tomography (SD-OCT) in CADASIL patients and showed significantly increased wall thicknesses as well as increased outer diameters of arterial and venous retinal vessels confirming histologic knowledge on pathophysiologic changes in vessel morphology^6^. Recently, a study by Fang and co-workers confirmed these results in a chinese CADASIL population. Moreover, they found a correlation between retinal vessel changes and findings in brain magnetic resonance imaging (MRI)^7^. Thus, retinal imaging appears to offer an additional perspective on CADASIL pathophysiology.

So far, OCT imaging has provided cross-sectional structural data of the retina and retinal vasculature without any information regarding blood flow. The standard retinal imaging techniques for visualizing retinal and choroidal perfusion are fluorescein angiography (FA) and indocyanine green angiography (ICGA). Both are invasive, two-dimensional en-face modalities that cannot show retinal blood flow depth-selectively. OCT-Angiography (OCT-A) represents a novel non-invasive, depth-selective modality that allows for visualization of retinal blood flow without dye injection^8^. OCT-A data on retinal blood flow or perfusion at the optic nerve head (ONH) can serve as additional diagnostic parameters not only in ocular disease but also in systemic disorders as recently shown in multiple sclerosis patients^9^.

Therefore, this study aims to evaluate macular retinal and choriocapillaris (CC) blood flow as well as blood flow at the ONH in CADASIL patients using OCT-A.

## Methods

### Patients

Eleven CADASIL patients belonging to seven families were recruited from the Department of Neurology at the University of Muenster Medical Center and were prospectively included for OCT-A measurements from March 2017 to August 2017. Nine of eleven patients had Notch3 mutations confirmed on genetic analysis and in two patients the disease was diagnosed based on vessel biopsy. Best-corrected visual acuity (BCVA) was tested. Patients with any of the following were excluded in the study: high myopia ( > −3 diopters), or any concomitant ocular disease. 21 age-matched healthy controls without any known medical conditions or ocular diseases served as a control group. Complete medical history was collected from all participants. This study was carried out in accordance with the relevant guidelines and regulations. Protocols were approved by the IRB of the Ärztekammer Westfalen-Lippe and University of Münster (2015-402-f-S). All subjects gave written informed consent.

### Optical coherence tomography angiography imaging

OCT-A imaging was conducted after pupillary dilation with a commercial SD OCT-system (AngioVue, RTVue XR Avanti SD-OCT, Optovue, Fremont, CA, USA). Images showing inadequate signal (signal strength index [SSI] < 50) or an OCT-A motion artifact score of three or four were excluded^10^. The device used in this study provides a scheme that defines en-face slabs according to a set of reference planes automatically segmented by the integrated software including the internal limiting membrane (ILM), the inner plexiform layer (IPL) and the retinal pigment epithlium (RPE) ref.^10^. Automatic segmentation was checked for accuracy by an experienced grader.

In the 3 × 3mm^2^ macula OCT-A image, the foveal avascular zone (FAZ) and the parafoveal vessel density (VD) of the superficial retinal plexus (SRP) and the deep retinal plexus (DRP) were measured using the software of the device.

**Figure 1:**
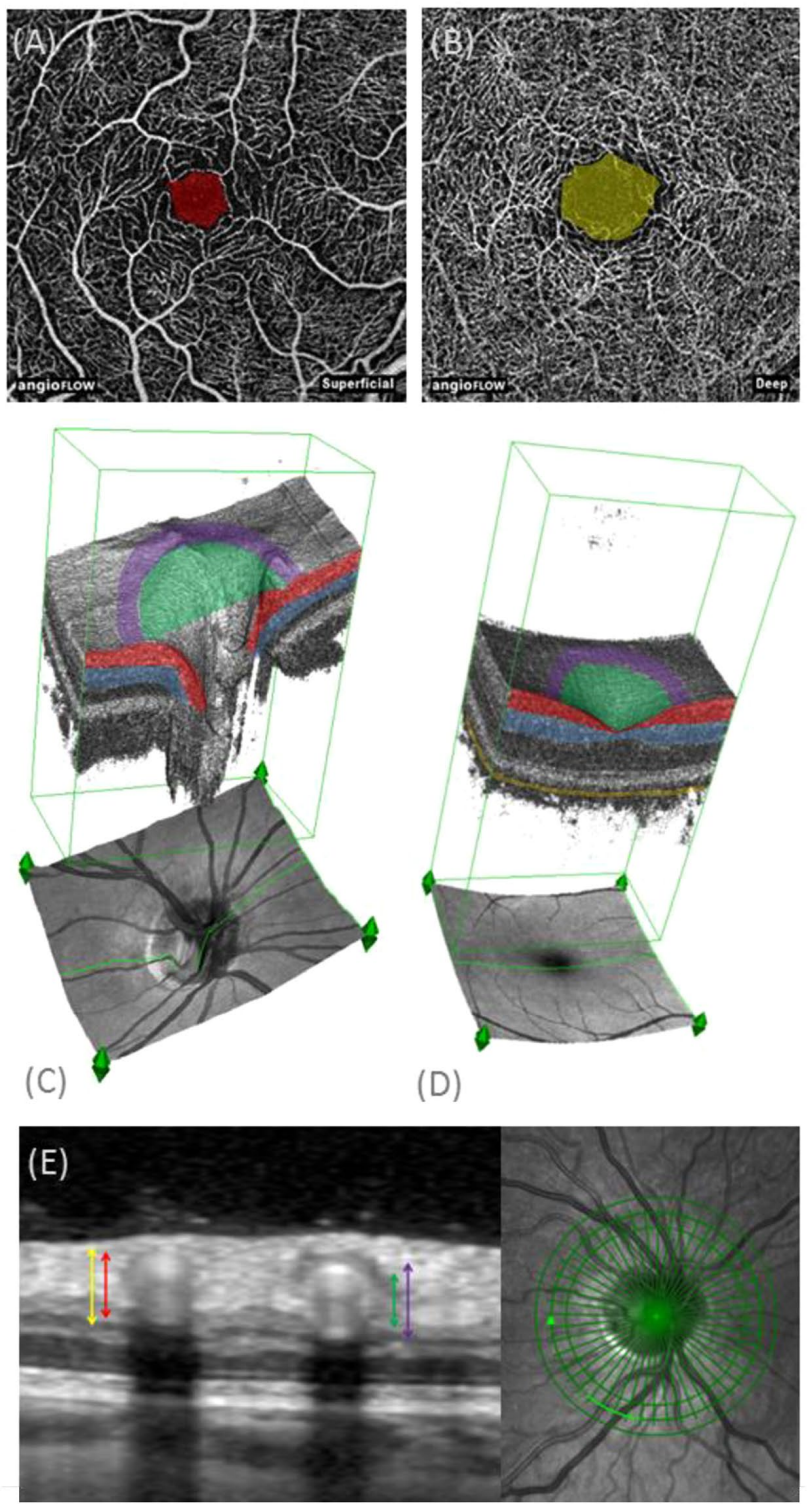
**(A)** Macular optical coherence tomography angiography (OCT-A) image of superficial retinal plexus with superimposed foveal avascular area (FAZs) (red). **(B)** Macular OCT-A image of deep retinal plexus with superimposed foveal avascular area (FAZd) area (yellow). **(C)** 3D-reconstruction of optic nerve OCT-A image with superimposed color coding representing segmentation layers and topical regions: radial peripapillary capillary (red), nerve head (red and blue), inside the disc (green), peripapillary region (purple). **(D)** 3D-reconstruction of macular OCT-A image with superimposed color coding representing segmentation layers and topical regions: superficial retinal layer (red), deep retinal layer (blue), choroidal layer (orange), parafoveal region (purple). **(E)** Combined simultaneous confocal scanning laser ophthalmoscopy (cSLO) and spectral-domain OCT. Detail of the circular OCT scan (left image) around the optic nerve head (right image) showing cross sections of two large peripapillary vessels with exemplary indications of venous (left) and arterial (right) outer (yellow, purple) and inner (red, green) diameter.

In the 4.5 × 4.5mm^2^ ONH OCT-A image, the VD of the radial peripapillary capillary layer and nerve head layer inside the disc and outside the disc were measured using the software of the device (Fig. 1).

**Figure 2:**
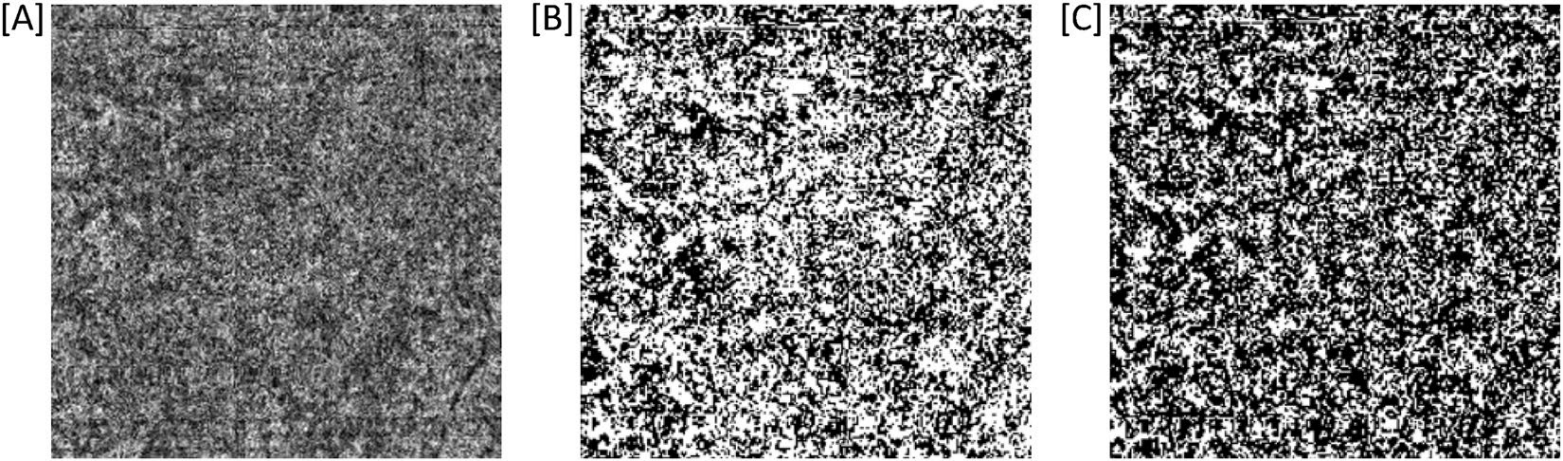
Exemplary en-face optical coherence tomography angiography (OCT-A) image of the choriocapillaris (CC) layer (30µm-slab between 30 µm and 60 µm below the inner retinal pigment epithelium reference). **(A)** Without image processing as used for CC decorrelation index analysis (average grey scale value of all pixels). **(B)** After Otsu thresholding as used for flow area analysis (percentage of the portion of white pixels against the whole scan area). **(C)** After Phansalkar thresholding as used for flow void analysis (proportion of white pixels accounted for by neighbouring white pixel regions greater than 10,000 µm^2^ [FV10000] and greater than 40,000 µm^2^ [FV40000]).

Three approaches were used to quantify CC blood flow (Fig. 2):

I. CC Decorrelation signal index: The standard setting of the software for CC imaging is a 30 µm-slab between 30 µm and 60 µm below the inner RPE reference. CC OCT-A image data were exported and further analyzed using the open access image analysis software ImageJ (Version 1.51n, National Institutes of Health, USA). Images were converted into grey scale values attributing each pixel to a value that represents the strength of the decorrelation signal. CC decorrelation signal index was defined as the average decorrelation value of all pixels in the en-face CC angiogram^11^.
II. Signal binarization: Each 304 × 304 pixels 8-bit images was binarized to calculate the percentage of black and white pixels^12^. The CC vascular flow area was defined as the percentage of the portion of white pixels against the whole scan area.
III. Analysis of signal voids: Automatic local thresholding was performed with the Phansalkar method using a radius of 15 pixels. The proportion of absent flow signal accounted for by signal voids greater than 10,000 µm^2^ (FV10000) and signal voids greater than 40,000 µm^2^ (FV40000) were evaluated^13^.

FAZ and macular VD data of the SRP and the DRP, CC data and ONH VD data of the superficial radial capillary layer and nerve head layer of CADASIL patients were compared to age-matched healthy control patients.

### Structural optical coherence tomography imaging

On cross-sectional peripapillary SD-OCT images, normal major retinal vessels appear as oval structures with four hyper-reflectivities stacked upon each other, the innermost and outermost originating from the inner (towards the vitreous) and outer (towards the RPE) sides of the vessel wall and a paired, frequently hourglass-shaped, hyper-reflectivity in the vessel lumen derived from the blood flow^14^. Using the method employed by Muraoka and co-workers, inner and outer vessel diameters of the two largest temporal retinal arteries (IDa, Oda) and veins (IDv, ODv) were measured vertically by a blinded grader using the built-in manufacturer’s software (Heidelberg Eye Explorer, Heidelberg Engineering,Germany) on a circular B-scan placed around the optic disc (diameter: 3.5 mm)^14^. Vessel wall thickness of arteries (VWa) and veins (VWv) was calculated as difference between outer and inner vessel diameter measurements (Fig. 1).

### Brain magnetic resonance imaging

MRI analysis was performed retrospectively to determine disease severity. All but two patients underwent MRI examination including Fluid-Attenuated-Inversion-Recovery (FLAIR) sequences within the last five years using different scanners with a field strength of 1.5- or 3-Tesla. White matter lesions were scored using Fazekas scale (total score range: 0–6)^15^. Additionally, the numbers of small infarcts were documented^16^. MRI analysis was performed by a single, experienced neuroradiologist blinded to OCT-A data.

### Statistical methods

OCT-A values and vessel diameter values are presented as mean ± standard deviation (SD). D’Agostino-Pearson normality test was performed to evaluate normality. Mann-Whitney-U-test (two-tailed) for not normal distributed values was used to compare OCT-A data and retinal vessel measurement data between patients and healthy controls. Values from both eyes were used for statistical comparisons between the groups. Statistical significance was defined as P < 0.001, considering a Bonferroni correction for multiple comparisons and the novelty of OCT-A imaging. OCT-A data of both eyes were correlated with MRI findings. The correlation was analyzed using Spearman’s correlation coefficient. Statistical testing was performed using Prism 7.02 (GraphPad Software nc., La Jolla, USA).

The datasets generated during and/or analysed during the current study are available from the corresponding author on reasonable request.

## Results

### Patients

21 eyes of eleven CADASIL patients (three male and eight female; mean age: 53.5 ± 10.7 years, range 38–74 years) were compared with 21 eyes of healthy controls (53.8 ± 11.5 years, range 28–77 years, p = 0.90). Mean disease duration was 14.0 ± 6.6 years. BCVA of healthy controls and CADASIL patients was LogMar 0.01 ± 0.04 and 0.02 ± 0.04 respectively. Five of eleven patients take antiplatelet agents in contrast to none of healthy controls. One eye suffered from refractive amblyopia. One patient had a history of unilateral central retinal artery occlusion and was excluded from vessel analysis.

### Optical coherence tomography angiography imaging

SSI of ONH OCT-A images was significantly lower in the CADASIL group in comparison with the healthy controls (controls: 70.11 ± 5.91, CADASIL: 62.24 ± 8.04, p = 0.0008). SSI of macular OCT-A images showed no significant difference between patients and controls (controls: 70.89 ± 6.05, CADASIL: 68.64 ± 5.89, p = 0.273).

VD of the DRP was significantly decreased in the CADASIL group (controls: 61.33 ± 1.93, 57.62 ± 2.46, p < 0.0001). All other macular and ONH OCT-A parameters were not significantly different between the two groups. CC OCT-A data showed no differences between CADASIL patients and healthy controls (Table 1).

**Table 1:** Data of optical coherence tomography angiography (OCT-A) parameters measured in control subjects versus CADASIL patients.

Variables	Controls (n = 21)	CADASIL (n = 21)	p-value
**Macular parameters**			
FAZs [mm^2^]	0.30 ± 0.11	0.27 ± 0.11	0.27
FAZd [mm^2^]	0.40 ± 0.14	0.35 ± 0.11	0.30
VDsM [%]	53.76 ± 4.87	53.25 ± 2.10	0.06
VDdM [%]	61.33 ± 1.93	57.62 ± 2.46	<0.0001
**Optic nerve head parameters**			
VDrID [%]	48.02 ± 9.41	45.54 ± 11.04	0.44
VDrPP [%]	64.01 ± 3.63	63.58 ± 1.93	0.84
VDnhID [%]	56.23 ± 4.73	52.61 ± 9.71	0.27
VDnhPP [%]	63.29 ± 2.34	63.86 ± 1.79	0.69
ODa [µm]	132.10 ± 16.16	141.80 ± 14.52	0.07
IDa [µm]	103.30 ± 13.96	108.50 ± 13.77	0.31
VWa [µm]	14.37 ± 2.75	16.65 ± 2.04	0.01
ODv [µm]	165.30 ± 13.73	167.00 ± 16.44	0.64
IDv [µm]	137.60 ± 14.58	140.50 ± 15.47	0.34
VWv [µm]	13.86 ± 2.63	13.29 ± 3.05	0.42
**Choriocapillaris [CC] parameters**			
CC decorrelation index [%]	103.30 ± 3.50	102.30 ± 3.67	0.26
Flow area [%]	45.16 ± 1.24	45.17 ± 1.25	0.96
FV10000 [µm^2^]	0.70 ± 0.04	0.65 ± 0.06	0.04
FV 40000 [µm^2^]	0.39 ± 0.07	0.35 ± 0.08	0.14

Foveal avascular zone of superficial retinal plexus (FAZs), FAZ of deep retinal plexus (FAZd), vessel density (VD) in superficial plexus of parafoveal region of central macula (VDsM), VD in deep plexus of parafoveal region of central macula (VDdM), VD in the radial peripapillary capillary layer of disc region (VDrID), VD in the nerve head layer of disc region (VDnhID), VD in the radial peripapillary capillary layer of peripapillary region (VDrPP), VD in the nerve head layer of peripapillary region (VDnhPP), outer diameter of artery (Oda), inner diameter of artery (Ida), arterial vessel wall (VWa), outer diameter of vein (ODv), inner diameter of vein (IDv), venous vessel wall (VWv), choriocapillaris (CC) decorrelation index, flow area, flow void area larger than 10,000 µm^2^ (FV10000), flow void area larger than 40,000 µm^2^ (FV40000).

### Structural optical coherence tomography imaging

Mean arterial and venous outer and inner diameters, as well as arterial and venous wall thickness were not significantly different between the two groups (Table 1).

### Brain magnetic resonance imaging examination

Mean Fazekas score was 4.22 ± 1.79. Infarcts were observed in 7/9 (77.8%) patients. Mean number of small infarcts was 8.67 ± 6.76. Fazekas scores and number of small infarcts in MRI did not correlate with decrease of perifoveal VD of DRP in OCT-A (p = 0.22; p = 0.28) and neither with any other OCT-A or OCT parameter measured.

## Discussion

In the past, scientists have continuously tried to benefit from the eye as a window to systemic conditions. Cerebral and retinal arterioles share a similar anatomy, physiology, and embryology and there is evidence for an association between retinal vessel changes and cerebral small vessel disease^17^. The aim of our study was to benefit from recent advances in *in-vivo* retinal imaging, namely the introduction of OCT-A, and to analyze macular perfusion and ONH perfusion in CADASIL patients using this recent technology. To our knowledge, this is the first study to use OCT-A in CADASIL patients. We found a significant decrease in macular vessel density in the deep retinal plexus in CADASIL patients compared to healthy controls.

Due to the two-dimensionality of conventional FA and ICGA, it has not been possible before to attribute findings specifically to the different retinal layers. Besides, FA is limited in its ability to resolve the depth of specific signal abnormalities, and cannot visualize the deeper retinal layers due to fluorescence blocked by superficial tissue. Similarly, ICGA cannot visualize the CC separatedly from the layers of the medium and large vessels of the choroid. For the first time, OCT-A offers depth-selective imaging of the DRP and the CC and provides valuable additional information over FA.

In 2006, Harju and co-workers reported a reduced retinal capillary blood flow in CADASIL using scanning laser doppler flowmetry. However, the method was restricted to a capillary diameter of approximately 100 µm and therefore, only large retinal vessels in proximity to the ONH were measured^18^. Notably, there is also some debate regarding the accuracy of this method. Our patients reported no visual symptoms and their visual acuity was normal. The reduced VD in the DRP does not appear to be of such severity that subjectively notable retinal damage had occurred. However, previous electrophysiologic studies provided evidence for subtle dysfunction in the retina in CADASIL patients^19^. Capillaries of the DRP are important for nutritional and oxygen support particularly at the border of the deep portion of the inner nuclear layer (INL) and outer plexiform layer (OPL), where the photoreceptor axon terminals form ribbon synapses with the horizontal cells and bipolar cells^20^. The area of the INL and OPL is located near a watershed zone between retinal and CC perfusion, where the oxygen level is significantly lower than that in the inner and outer retinal layer^21^. Hypoperfusion in the DRP may cause nutritional deficiency in the synaptic connections, possibly explaining the previously reported electrophysiologic impairments in CADASIL patients^19^.

The small retinal capillaries lack smooth muscle cells, the preferential site of damage in CADASIL. Thus, one may argue that pathologic changes can obviously be expected in large retinal vessels but not in small retinal capillaries. Yet, Notch3, a cell surface receptor, is not only expressed in vascular smooth muscle cells but also in pericytes^1,22^. Haritoglou *et al*. found pericyte alterations in retinal vasculature in histopathological examinations of CADASIL patients^4^. Using light microscopy, retinal capillaries showed pericytes with swollen nuclei. Electron microscopy additionally revealed pericyte degeneration^4^. Also in autopsy brains and skin-muscle biopsies, degeneration and loss of pericytes in capillary vessels were described^23^. The reduced VD in the DRP of CADASIL patients found in our study may be the pathologic expression of Notch3 mutations in pericytes. Retinal pericytes are essential constituents of the blood retina barrier and fulfill important functions to maintain vessel homeostasis. Interestingly, the most important retinal pathology associated with reduced pericyte coverage is diabetic retinopathy. Similarly to our data in CADASIL patients, a characteristic affection of the DRP has also been described for diabetic retinopathy^24^. Several authors reported that in patients with diabetic retinopathy the DRP is affected earlier by diabetic alterations than the SRP^25^. Possibly in a study with a longer follow-up or with CADASIL patients having longer disease duration, a VD decrease might also be detectable in the SRP as it is in diabetic retinopathy.

In pathological specimens of CADASIL patients, Miao and co-workers found a thickening of the arterial wall leading to luminal stenosis^26^. An arterial outer diameter and an arterial wall thickness increase were reported based on manual measurements in OCT-scans in Fang *et al*. and Alten *et al*.^6,7^. Correspondingly, a similar trend can be observed in our data. As in Alten *et al*., we found no significant difference for inner arterial diameter. Literature reports an increase in venous outer and inner vessel diameter as reflected in our data^6,7^.

Recently, Fang and co-workers reported a reduced subfoveal choroidal thickness in CADASIL patients using enhanced depth (EDI) OCT^7^. Notably, subfoveal choroidal thickness is highly variable and influenced by numerous variables such as arterial hypertension or circadian fluctuations. Apart from a single case report describing irregular choroidal filling on FA, this is the only study showing choroidal changes in CADASIL patients^5^. In 2014, Alten and colleagues performed an extensive imaging of the choroid in CADASIL using EDI-OCT volume scans and ICGA. No differences were found compared to control subjects^6^. However, neither ICGA nor EDI-OCT imaging can image the CC layer depth-selectively and neither can offer quantitative measurements of CC flow. Our presented OCT-A data includes three approaches of a selective CC flow quantification. All show no significant difference between CADASIL and controls suggesting that vessel alterations in the back of the eye due to CADASIL are restricted to retinal vessels. Previous histologic data revealed that ocular vessel pathologies in CADASIL patients are limited to retinal vessels only, while choroidal vessels are unaffected^4^.

Findings in MRI FLAIR sequences did not correlate to OCT-A findings. Interestingly, we saw OCT-A changes in the DRP even in those CADASIL patients with only very mild MRI FLAIR findings. Future studies should include diffusion weighted-MRI and high-field 7 T MRI to draw further conclusions regarding the association of cerebral and retinal capillary changes in CADASIL and other small vessel diseases^16^.

Obviously, the small number of subjects included in the study precludes any definitive interpretation. Yet, CADASIL is a rare disease. In those patients included, diagnosis was confirmed by genetic testing and vessel biopsy. In the CADASIL group, SSI values were significantly lower in ONH OCTA images compared to healthy controls, while SSI values in macular OCTA images were not. ONH OCTA measurements require a higher level of cooperation due to a lateral gaze participants have to hold. CADASIL patients’ cooperation may be reduced due to cognitive impairment caused by their pathology. Furthermore, the clinical stage of the disease was heterogeneous within the study group. The OCT-A device used in this study differentiates between a SRP and a DRP. There is ongoing debate regarding nomenclature and segmentation boundaries for detailed 3-dimensional retinal vascular anatomy by OCT-A. Furthermore, projection-resolved OCT-A algorithms are being developed that improve depth resolution by removing projection artifacts while retaining flow signals from real blood vessels in deeper layers.

In conclusion, CADASIL patients showed a distinct reduction in VD in the DRP which may reflect pericyte dysfunction in retinal capillaris due to Notch3 mutations. In the future, a decrease in VD of retinal capillaris detected in OCT-A might serve as an additional disease marker in CADASIL and other small vessel diseases.
